# Supplementary material for: DNA tensiometer reveals catch-bond detachment kinetics of kinesin-1, -2, and -3
Source: eLife. 2026 Jul 20;14:RP108837. doi: 10.7554/eLife.108837 (PMC13384492; doi:10.7554/eLife.108837)
Supplement: Peer review history

# Editor report: eLife Assessment

**DOI:** 10.7554/eLife.108837.4.sa0

**Authors:** Kerry Bloom

**Affiliations:** The University of North Carolina at Chapel Hill United States

**Keywords:** Convincing, Valuable

The authors use **convincing** methodology to investigate the detachment and reattachment kinetics of kinesin-1, 2 and 3 motors against loads oriented parallel to the microtubule. The conclusions drawn from the **valuable** experiments as well as the overall interpretation of the results are fully supported by the presented data.

# Referee report: Reviewer #1 (Public review)

**DOI:** 10.7554/eLife.108837.4.sa1

[Editors' note: this version has been assessed by the Reviewing Editor without further input from the original reviewers. The authors have addressed the comments raised in the previous round of review.]

Summary:

Noell et al have presented a careful study of the dissociation kinetics of Kinesin (1,2,3) classes of motors moving in-vitro on a microtubule. These motors move against the opposing force from a ~1 micron DNA strand (DNA tensiometer) that is tethered to the microtubule and also bound to the motor via specific linkages (Fig 1A). Authors compare the time for which motors remain attached to the microtubule when they are tethered to the DNA, versus when they are not. If the former is longer, the intepretation is that the force on the motor from the stretched DNA (presumed to be working solely along the length of the microtubule) causes the motor's detachment rate from the microtubule to be reduced. Thus, the specific motor exhibits "catch-bond" like behaviour.

Strengths:

The motivation is good - to understand how kinesin competes against dynein through the possible activation of a catch bond. Experiments are well done and there is an effort to model the results theoretically.

Weaknesses from original round of review:

The motivation of these studies is to understand how kinesin (1/2/3) motors would behave when they are pitted in a tug of war against dynein motors as they transport cargo in bidirectional manner on microtubules. Earlier work on dynein and kinesin motors using optical tweezers has suggested that dynein shows catch bond phenomenon, whereas such signatures were not seen for kinesin. Based on their data with DNA tensiometer, the authors would like to claim that (i) Kinesin1 and kinesin2 also show catch-bonding and (ii) The earlier results using optical traps suffer from vertical forces, which complicates the catch-bond interpretation.

# Referee report: Reviewer #2 (Public review)

**DOI:** 10.7554/eLife.108837.4.sa2

Summary:

To investigate the detachment and reattachment kinetics of kinesin-1, 2 and 3 motors against loads oriented parallel to the microtubule, the authors used a DNA tensiometer approach comprising a DNA entropic spring attached to the microtubule on one end and a motor on the other. They found that for kinesin-1 and kinesin-2 the dissociation rates at stall were smaller than the detachment rates during unloaded runs. With regard to the complex reattachment kinetics found in the experiments, the authors argue that these findings were consistent with a weakly-bound 'slip' state preceding motor dissociation from the microtubule. The behavior of kinesin-3 was different and (by the definition of the authors) only showed prolonged "detachment" rates when disregarding some of the slip events. The authors performed stochastic simulations which recapitulate the load-dependent detachment and reattachment kinetics for all three motors. They argue that the presented results provide insight into how kinesin-1, -2 and -3 families transport cargo in complex cellular geometries and compete against dynein during bidirectional transport.

Strengths:

The present study is timely, as significant concerns have been raised previously about studying motor kinetics in optical (single-bead) traps where significant vertical forces are present. Moreover, the obtained data are of high quality and the experimental procedures are clearly described.

# Referee report: Reviewer #3 (Public review)

**DOI:** 10.7554/eLife.108837.4.sa3

Summary:

Several recent findings indicate that forces perpendicular to the microtubule accelerate kinesin unbinding, where perpendicular and axial forces were analyzed using the geometry in a single-bead optical trapping assay (Khataee and Howard, 2019), comparison between single-bead and dumbbell assay measurements (Pyrpassopoulos et al., 2020), and comparison of single-bead optical trap measurements with and without a DNA tether (Hensley and Yildiz, 2025).

Here, the authors devise an assay to exert forces along the microtubule axis by tethering kinesin to the microtubule via a dsDNA tether. They compared the behavior of kinesin-1, -2, and -3 when pulling against the DNA tether. In line with previous optical trapping measurements, kinesin unbinding is less sensitive forces when the forces are aligned with the microtubule axis. Surprisingly, the authors find that both kinesin-1 and -2 detach from the microtubule more slowly when stalled against the DNA tether than in unloaded conditions, indicating that these motors act as catch bonds in response to axial loads. Axial loads accelerate kinesin-3 detachment. However, kinesin-3 reattaches quickly to maintain forces. For all three kinesins, the authors observe weakly-attached states where the motor briefly slips along the microtubule before continuing a processive run.

Strengths:

These observations suggest that the conventional view that kinesins act as slip bonds under load, as concluded from single-bead optical trapping measurements where perpendicular loads are present due to the force being exerted on the centroid of a large (relative to the kinesin) bead, need to be reconsidered. Understanding the effect of force on the association kinetics of kinesin has important implications for intracellular transport, where the force-dependent detachment governs how kinesins interact with other kinesins and opposing dynein motors (Muller et al., 2008; Kunwar et al., 2011; Ohashi et al., 2018; Gicking et al., 2022) on vesicular cargoes.

# Author comment: Author response

**DOI:** 10.7554/eLife.108837.4.sa4

**Authors:** Crystal R Noell, Tzu-Chen Ma, Rui Jiang, Scott A McKinley, William O Hancock

**Affiliations:** Pennsylvania State University University Park United States; Pennsylvania State University University Park United States; Pennsylvania State University University Park United States; Tulane University New Orleans United States; Pennsylvania State University University Park United States

The following is the authors’ response to the current reviews.

**Reviewer #1 (Public review):**

I am not fully convinced about the responses from authors, so I would like to retain my original assessment of the paper. The same may be made available for public viewing, along with the responses of the authors. Readers can go through both and form their opinion.

Unfortunately, this response from Reviewer 1 impacted the Assessment Statement but did not provide specific points for us to address. In the first round, the concerns of Reviewer 1 were: (1) the validity of the WLC prediction; (2) the claim that catch-bond measurements are generally made with superstall loads; (3) the role of vertical forces for dynein and a question about the orientation of the forces for kinesin; and (4) a request that we repeat the study using dynein. In rereading our responses to points 2-4 following our first revision, we felt that there were no unresolved issues around those points that affect our conclusions in any way. However, for point 1 regarding the validity of the WLC prediction, we had responded only in the reviewer response letter, and both reviewer 2 and the editors felt that there were points that we had addressed in the response letter that should be incorporated into the revised manuscript. Therefore, to clarify Reviewer 1’s question, we revised the text to address why we were justified to approximate the dsDNA force-extension curve using a WLC model with a 50 nm persistence length and why the precise shape of the force-extension curve has no impact on our conclusions.

**Reviewer #2 (Public review):**

The authors extensively entered into a scientific debate with the reviewers in their Response Letter. This led to a few changes and some (limited) new data in the manuscript. This is great and did improve the manuscript.

However, in the view of this reviewer, (i) a significant number of responses fall short of actually addressing the concerns of the three reviewers (e.g. wrt using the same kinesin-1 neck-coil domains for all motors) and or (ii) a significant number of arguments now only occur in the response letter but not in the manuscript. The authors may check themselves critically for both. In principle, each longer discussion in the response letter warrants mentioning the appropriate facts and arguments in the main text of the manuscript.

Based on this feedback, the first change we made was to rewrite the section justifying our choice of using a common coiled-coil dimerization domain for the three motors. Secondly, we went through our responses to all three reviewers to identify any instances where we either didn’t fully address the reviewer concerns or we provided arguments in the response letter but did not add corresponding text in the manuscript.

**Reviewer #3 (Public review):**

The authors attribute the differences in the behaviour of kinesins when pulling against a DNA tether compared to an optical trap to the differences in the perpendicular forces. However, the compliance is also much different in these two experiments. The optical trap acts like a ~ linear spring with stiffness ~ 0.05 pN/nm. The dsDNA tether is an entropic spring, with negligible stiffness at low extensions and very high compliance once the tether is extended to its contour length (Fig. 1B). The effect of the compliance on the results is not fully considered in the manuscript.

In our first revision we added a paragraph in the ‘Geometry Calculations section of the Supplementary Methods addressing the dsDNA stiffness and comparing it to an optical trap. We considered moving this paragraph to the main text but decided against it because we felt it interrupted the flow of the Discussion. Instead, we expanded and clarified this paragraph to more specifically address the stiffness question. The paragraph with revised text now reads as follows:

“Another consideration when comparing the DNA tensiometer to optical trap measurements is the relative stiffness of the trap and dsDNA. Optical traps stiffnesses are generally in the range of 0.05 pN/nm [13,14]. To calculate the predicted stiffness of the dsDNA spring, we computed the slope of theoretical force-extension curve in Fig. 1B. The stiffness is highly nonlinear and is <0.001 pN/nM below 650 nm extension. We compare motor performance under this low stiffness regime to the unloaded case in Fig. 3. In contrast, at the predicted stall force of 6 pN (960 nm extension), the dsDNA stiffness is ~0.2 pN/nm, which is stiffer than most optical traps, but it is similar to the estimated 0.3 pN/nm stiffness of kinesin motors themselves [13,14]. An 8 nm step at the 0.2 pN/nm stiffness of the dsDNA leads to a 1.6 pN jump in force and at the 0.05 pN/nm stiffness of an optical trap leads to a 0.4 pN jump in force; this is important because it means that in both cases the motors are likely dynamically stepping at stall. Because both experimental approaches allow for dynamic stepping at stall and because the stiffnesses of the instrument in both cases are less than the motor stiffness, there is no reason to expect that differences in stiffness between optical traps and the dsDNA spring lead to different motor detachment kinetics.”

In the main text, we now address this compliance point in the ‘Comparison to previous work’ section of the Discussion:

“stiffness differences are an unlikely explanation because at stall the stiffness of the DNA tether (~4 fold stiffer than optical tweezer) is still sufficiently low to allow for dynamic motor stepping at stall, and in any case it is still below the estimated motor stiffness (see Geometry Calculations in Supplementary methods).”.

There were two points the reviewer felt we had sufficiently addressed. They were presented in the second review as a reiteration of the first review comments with a sentence appended, and are reproduced here. We added no new text based on these two points:

In the single-molecule extension traces (Fig. 1F-H; S3), the kinesin-2 traces often show jumps in position at the beginning of runs (e.g. the four runs from ~4-13 s in Fig. 1G). These jumps are not apparent in the kinesin-1 and -3 traces. What is the explanation? Is kinesin-2 binding accelerated by resisting loads more strongly than kinesin-1 and -3? In their response, the authors provide an explanation of the appearance of jumps due to limited imaging speeds. The authors state that the qualitative difference in the kinesin-2 traces compared to the kinesin-1 and -3 traces may be due to the specific rebinding kinetics of kinesin-2.

When comparing the durations of unloaded and stall events (Fig. 2), there is a potential for bias in the measurement, where very long unloaded runs cannot be observed due to the limited length of the microtubule (Thompson, Hoeprich, and Berger, 2013), while the duration of tethered runs is only limited by photobleaching. Was the possible censoring of the results addressed in the analysis? The authors addressed this concern by applying a Markov model to estimate the duration parameter.

There was one final point from Reviewer 3 in the first round of reviews that we had addressed in the reviewer response (and that the reviewer was satisfied with), but we did not incorporate into the manuscript. Based on the suggestion from Reviewer 2 and the editors that we incorporate more from our responses to reviewers into the manuscript, we added new text on this point. That point (with the new sentence in the second review underlined), our response from first revision, and our response for this second revision are given below:

The mathematical model is helpful in interpreting the data. To assess how the "slip" state contributes to the association kinetics, it would be helpful to compare the proposed model with a similar model with no slip state. Could the slips be explained by fast reattachments from the detached state? In their response, the authors addressed this question by explaining that a three-state model is required to model the recovery time distributions.

In the model, the slip state and the detached states are conceptually similar; they only differ in the sequence (slip to detached) and the transition rates into and out of them. The simple answer is: yes, the slips could be explained by fast reattachments from the detached state. In that case, the slip state and recovery could be called a “detached state with fast reattachment kinetics”. However, the key data for defining the kinetics of the slip and detached states is the distribution of Recovery times shown in Fig. 4D-F, which required a triple exponential to account for all of the data. If we simplified the model by eliminating the slip state and incorporating fast reattachment from a single detached state, then the distribution of Recovery times would be a single-exponential with a time constant equivalent to t_1_, which would be a poor fit to the experimental distributions in Fig. 4D-F.

Reviewer 3 noted that they were satisfied with our explanation of this point. However, based on Reviewer 2’s suggestion that we incorporate more of our responses into the text of the manuscript, we added the following clarification point in the model section of the Results:

“We note that recapitulating the tri-exponential restart time distribution in Figure 4D-F required this slip/detached formulation and that lumping all events into a single detached state resulted in a single-exponential distribution of recovery times.”
